# RNAseq Analysis of Endornavirus-Infected vs. Endornavirus-Free Common Bean (*Phaseolus vulgaris*) Cultivar Black Turtle Soup

**DOI:** 10.3389/fmicb.2016.01905

**Published:** 2016-11-29

**Authors:** Surasak Khankhum, Noa Sela, Juan M. Osorno, Rodrigo A. Valverde

**Affiliations:** ^1^Department of Plant Pathology and Crop Physiology, Louisiana State University Agricultural CenterBaton Rouge, LA, USA; ^2^Department of Plant Pathology and Weed Research, The Volcani Center-AROBet-Dagan, Israel; ^3^Department of Plant Sciences, North Dakota State UniversityFargo, ND, USA

**Keywords:** common bean, endornavirus, RNA sequencing, *Phaseolus vulgaris endornavirus 1*, *Phaseolus vulgaris endornavirus 2*

## Introduction

Common bean (*Phaseolus vulgaris* L.) is the most important grain legume for direct human consumption worldwide and represents a rich source of protein, vitamins, minerals, and fiber (Broughton et al., 2003). The recent sequencing of the common bean genome, together with the availability of genomic and transcriptomic data have provided useful information to common bean breeders that will help in the development of genotypes with desirable characteristics (Schmutz et al., 2014; Vlasova et al., 2016).

Endornaviruses are persistent viruses with a non-encapsidated RNA genome that ranges from 9.8 to 17.6 kb, infect plants, fungi, and oomycetes, are transmitted only via gametes, and do not cause apparent symptoms (Stielow et al., 2011; Fukuhara and Gibbs, 2012). Although endornaviruses have been reported in several economically important plant species, little is known about the effect they have on their hosts. One of the major obstacles to study their effect to the host is the lack of a transmission method. In plants, endornaviruses do not move from cell to cell and spread only during cell division.

Recently, Khankhum et al. (2015) reported that most common bean genotypes of Mesoamerican origin are double-infected with *Phaseolus vulgaris endornavirus 1* (PvEV1) and *Phaseolus endornavirus 2* (PvEV2); in contrast, genotypes of Andean origin are often endornavirus-free. Black Turtle Soup (BTS), a cultivar of Mesoamerican origin has been reported to be double-infected by these two endornaviruses (Okada et al., 2013). A BTS endornavirus-free selection (BTS−), obtained from an endornavirus-infected BTS (BTS+) seed lot has been reported by Okada et al. (2013). To establish the bases for future research on the role that endornaviruses play in the common bean plant, and the effect these viruses have on the host gene expression, we conducted RNAseq on two BTS lines: one endornavirus-infected and the other endornavirus-free.

## Value of the data

Currently, there are no sources of gene annotation for any organism infected with endornaviruses. This information will be helpful in determining the nature of the symbiotic interaction between endornaviruses and their host; more specifically between Mesoamerican common bean and PvEV1 and PvEV2.

These data may help to identify relevant genes in common bean that are differentially expressed under endornavirus infections.

## Materials and methods

### Library preparation and transcriptome sequencing

Seeds from the BTS− selection and seeds from a BTS+ plant obtained in previous investigations (Okada et al., 2013) were increased at least three generations by self-pollination. Crosses using the BTS+ selection as male and the BTS− as female were conducted in the greenhouse facilities of the Department of Plant Sciences, North Dakota State University, Fargo, ND. From the F_1_ generation, a plant double-infected with PvEV1 and PvEV2 designated BTS+ 3 was selected and increased two generations. The original BTS− line was increased two generations and designated BTS− 4. For the detection of the two viruses in the plants selected for the RNAseq, we used two methods reported in previous investigations, electrophoretic analysis of extracted viral dsRNA and RT-PCR using specific primers for each virus (Khankhum et al., 2015, 2016). Seeds of each line were planted under controlled temperature (25°C) and light (16 h photoperiod) conditions. Three weeks after planting, 100 mg of leaf tissue (trifoliate leaves) was collected, placed in a 1.5 ml nuclease-free microcentrifuge tube, and immediately submerged in liquid nitrogen. Samples were kept at −70°C until ready for RNA extraction. Total RNA was extracted following the extraction procedure of the Spectrum™ Plant Total RNA Kit (Sigma-Aldrich, St. Louis, MO). Collected leaf tissues were ground in liquid nitrogen using a micro-pestle. To eliminate residual DNA contamination, the RNA was DNase treated using the On-Spin Column DNase I Kit (MO BIO Laboratory, Inc., Carlsbad, CA) following the manufacturers' directions. Total RNA was eluted out from the column using nuclease-free water. The quantity and quality of the RNA was determined using an Agilent 2100 Bioanalyzer (Agilent Technologies, Santa Clara, CA). Samples were placed in RNAstable® (Biomatrica Inc., San Diego, CA) tubes and shipped for sequencing. RNA sequencing was conducted by SeqMatic (SeqMatic, Fremont, CA). A total of six RNA libraries, three from individual plants of BTS− 4 and three from individual plants of BTS+ 3, were prepared using Illumina TruSeq Stranded Total RNA Library Prep Kit (Illumina, Diego, CA) and sequenced using the Illumina Hiseq2500 platform to generating 50 bp single-end reads.

### Bioinformatics analysis

The reference genome of common bean (*P. vulgaris*) version 1.0 (Schmutz et al., 2014) was downloaded from the Phytozome website (Goodstein et al., 2012). Six RNAseq libraries of BTS common bean, three double-infected with PvEV1 and PvEV2 and three endornavirus-free were mapped to the reference genome using bowtie software (Langmead and Salzberg, 2012). Quantification of the transcript expression was conducted using RSEM method (RNA-Seq by Expectation Maximization) (Li and Dewey, 2011). Differential expression analysis was done using R bioconductor package edgeR (Robinson et al., 2010). To associate sequences and gene expression data with biological functions, gene ontology (GO) distribution analysis was conducted using Blast2GO (Conesa et al., 2005).

## Results

**Figure 1:**
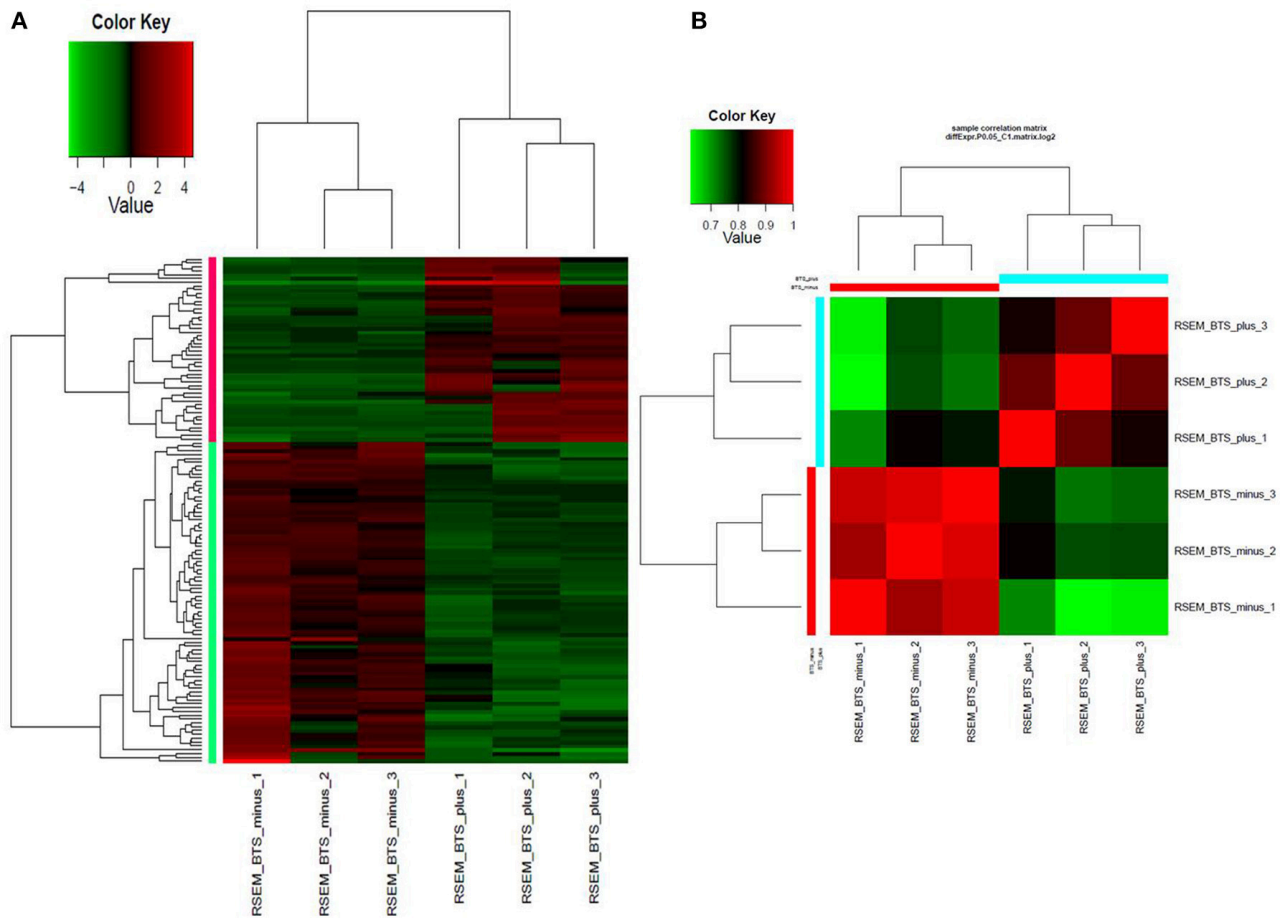
**(A)** Heatmap of normalized expression matrix of the six RNAseq libraries. Highly expressed genes are in red while low expressed genes are in green. **(B)** Heatmap of the Pearson correlation between the expression levels of differentially expressed genes. Red color marks highly correlated samples while green color marks low correlation. BTS_minus, endornavirus-free sample; BTS_plus, endornavirus-infected sample.

Differential expression analysis of RNAseq data revealed that a total of 132 genes were differentially expressed. In the endornavirus-infected line 84 genes were down-regulated while 48 genes up-regulated (Supplementary Tables 1, 2). Figures 1A,B shows a visual reference of the differentially expressed gene vs. samples heatmap and Pearson correlation heatmap. GO distribution data on up-regulated and down-regulated genes is provided as excel files in Data Sheets 1 and 2 respectively in Supplementary Material. Gene ontology distribution show that oxidation-reduction processes were the main process associated with endornavirus infection. Reduction–oxidation (redox) changes have been reported to be associated with plant response to pathogen infection (Frederickson Matika and Loake, 2014), environmental stresses, development, and acclimation (Dietz, 2014; Dietz et al., 2016; Carmody et al., 2016). Data Sheets 3–5 contain excel files with expression levels, *p*-values, and FPKM (fragments per kilobase of transcript per million mapped reads) values respectively for all genes of the virus-infected and virus-free plants.

### Direct link to deposited data and information to users

Raw reads were deposited into the NCBI Sequence Read Archive (SRA) database (https://www.ncbi.nlm.nih.gov/sra/srp090495) under accession SRP090495.

## Authors contributions

RV: conceived the idea and selected the original endornavirus-free BTS plant. SK: conducted the experiments; NS: performed the bioinformatics analysis of the data; JO: conducted the BTS crosses and contributed to the idea; RV, NS: wrote the manuscript. All authors contributed to the review of the manuscript.

## Funding

This research was conducted with partial support from research grant No. US-4725-14 F from BARD, the United States—Israel Binational Agricultural Research and Development Fund.

### Conflict of interest statement

The authors declare that the research was conducted in the absence of any commercial or financial relationships that could be construed as a potential conflict of interest.

## Abbreviations

BARD: Binational Agricultural Research and Development
BTS: Black Turtle Soup
BTS−: Black Turtle Soup endornavirus-free
BTS+: Black Turtle Soup endornavirus-infected
FPKM: Fragments per kilobase of transcript per million mapped reads
GO: Gene ontology
PvEV1: Phaseolus vulgaris endornavirus 1
PvEV2: Phaseolus vulgaris endornavirus 2.
