# Supplementary material for: RNAseq Analysis of Endornavirus-Infected vs. Endornavirus-Free Common Bean (Phaseolus vulgaris) Cultivar Black Turtle Soup
Source: Front Microbiol. 2016 Nov 29;7:1905. doi: 10.3389/fmicb.2016.01905 (PMC5126043; doi:10.3389/fmicb.2016.01905)
Supplement: Supplementary file 1 [file Table1.DOCX]

**Khankhum et al., 2016**

**Supplementary material**

**Table 1. Black Turtle Soup common bean genes up-regulated during endornavirus infection.**

| **Transcript id** | **Arabidopsis homolog** | **Gene name** |
| --- | --- | --- |
| Phvul.006G156100.1 | AT1G78950.1 | Terpenoid cyclases family protein |
| Phvul.006G151100.1 | AT4G04880.1 | adenosine/AMP deaminase family protein |
| Phvul.011G037100.1 | AT4G38840.1 | SAUR-like auxin-responsive protein family |
| Phvul.001G260600.1 | AT1G67480.1 | Galactose oxidase/kelch repeat superfamily protein |
| Phvul.001G158000.1 | ATCG00770.1 | ribosomal protein S8 |
| Phvul.001G155100.1 | Na | na |
| Phvul.008G032200.1 | AT5G39090.1 | HXXXD-type acyl-transferase family protein |
| Phvul.007G049100.1 | AT4G21410.1 | cysteine-rich RLK (RECEPTOR-like protein kinase) 29 |
| Phvul.008G277600.1 | AT3G06920.1 | Tetratricopeptide repeat (TPR)-like superfamily protein |
| Phvul.008G241700.1 | AT1G65840.1 | polyamine oxidase 4 |
| Phvul.L006900.1 | AT5G14180.1 | Myzus persicae-induced lipase 1 |
| Phvul.006G174000.1 | na | na |
| Phvul.008G279800.1 | AT1G55850.1 | cellulose synthase like E1 |
| Phvul.009G255400.1 | na | na |
| Phvul.007G036500.1 | AT2G01290.1 | ribose-5-phosphate isomerase 2 |
| Phvul.006G188500.1 | na | na |
| Phvul.L004800.1 | AT1G21690.1 | ATPase family associated with various cellular activities (AAA) |
| Phvul.008G279600.1 | AT1G55850.1 | cellulose synthase like E1 |
| Phvul.007G051600.1 | AT3G04290.1 | Li-tolerant lipase 1 |
| Phvul.004G012800.1 | AT3G14470.1 | NB-ARC domain-containing disease resistance protein |
| Phvul.003G140800.1 | AT5G57150.1 | basic helix-loop-helix (bHLH) DNA-binding superfamily protein |
| Phvul.003G223600.1 | AT5G52020.1 | Integrase-type DNA-binding superfamily protein |
| Phvul.006G000300.1 | AT4G22120.1 | ERD (early-responsive to dehydration stress) family protein |
| Phvul.005G047100.1 | AT5G50140.1 | Ankyrin repeat family protein |
| Phvul.005G053500.1 | AT1G12740.1 | cytochrome P450, family 87, subfamily A, polypeptide 2 |
| Phvul.004G054900.1 | AT5G14180.1 | Myzus persicae-induced lipase 1 |
| Phvul.008G227000.1 | AT2G39920.2 | HAD superfamily, subfamily IIIB acid phosphatase |
| Phvul.005G046600.1 | AT1G03670.1 | ankyrin repeat family protein |
| Phvul.004G054800.1 | AT5G14180.1 | Myzus persicae-induced lipase 1 |
| Phvul.008G030300.1 | na | na |
| Phvul.008G029400.1 | AT1G03940.1 | HXXXD-type acyl-transferase family protein |
| Phvul.004G123200.1 | AT5G62170.1 | na |
| Phvul.005G077000.1 | AT5G59400.1 | na |
| Phvul.011G089800.1 | AT1G08230.2 | Transmembrane amino acid transporter family protein |
| Phvul.008G227100.1 | AT3G55780.1 | Glycosyl hydrolase superfamily protein |
| Phvul.008G276200.1 | AT3G13080.1 | multidrug resistance-associated protein 3 |
| Phvul.005G080500.1 | na | na |
| Phvul.007G051000.1 | AT4G05200.1 | cysteine-rich RLK (RECEPTOR-like protein kinase) 25 |
| Phvul.006G200600.1 | AT1G72680.1 | cinnamyl-alcohol dehydrogenase |
| Phvul.004G150700.1 | na | na |
| Phvul.005G053600.1 | AT1G12740.1 | cytochrome P450, family 87, subfamily A, polypeptide 2 |
| Phvul.002G170300.1 | AT2G03090.1 | expansin A15 |
| Phvul.003G143900.1 | AT2G18950.1 | homogentisate phytyltransferase 1 |
| Phvul.009G111800.1 | AT3G16360.2 | HPT phosphotransmitter 4 |
| Phvul.009G122800.1 | AT1G77670.1 | Pyridoxal phosphate (PLP)-dependent transferases superfamily protein |
| Phvul.007G050900.1 | AT4G05200.1 | cysteine-rich RLK (RECEPTOR-like protein kinase) 25 |
| Phvul.008G277100.1 | AT3G13080.1 | multidrug resistance-associated protein 3 |
| Phvul.005G053400.1 | AT1G12740.1 | cytochrome P450, family 87, subfamily A, polypeptide 2 |

*na-indicates gene without annotations or without Arabidopsis homolog*
