# Supplementary material for: RNAseq Analysis of Endornavirus-Infected vs. Endornavirus-Free Common Bean (Phaseolus vulgaris) Cultivar Black Turtle Soup
Source: Front Microbiol. 2016 Nov 29;7:1905. doi: 10.3389/fmicb.2016.01905 (PMC5126043; doi:10.3389/fmicb.2016.01905)
Supplement: Supplementary file 2 [file Table2.DOCX]

Khankhum et al., 2016

Supplementary material

**Table 2. Black Turtle Soup common bean genes down-regulated during endornavirus infection.**

| **Transcript id** | **Arabidopsis homolog** | **Gene name** |
| --- | --- | --- |
| Phvul.003G024400.1 | na | na |
| Phvul.009G258500.1 | AT2G46820.1 | photosystem I P subunit |
| Phvul.011G100800.1 | AT5G06570.1 | alpha/beta-Hydrolases superfamily protein |
| Phvul.008G237400.1 | AT5G37600.1 | glutamine synthase clone R1 |
| Phvul.005G181300.1 | AT4G37850.1 | basic helix-loop-helix (bHLH) DNA-binding superfamily protein |
| Phvul.007G121100.1 | AT3G04550.1 | na |
| Phvul.008G164000.1 | AT4G08300.1 | nodulin MtN21 /EamA-like transporter family protein |
| Phvul.005G160800.1 | AT1G17710.1 | Pyridoxal phosphate phosphatase-related protein |
| Phvul.006G147900.1 | AT1G11910.1 | aspartic proteinase A1 |
| Phvul.006G185900.1 | AT4G03510.1 | RING membrane-anchor 1 |
| Phvul.010G139800.1 | AT1G55180.1 | phospholipase D alpha 4 |
| Phvul.004G097800.1 | na | na |
| Phvul.008G048400.1 | AT1G71460.1 | Pentatricopeptide repeat (PPR-like) superfamily protein |
| Phvul.003G194000.1 | AT2G45350.1 | Pentatricopeptide repeat (PPR) superfamily protein |
| Phvul.011G186600.1 | AT5G61750.1 | RmlC-like cupins superfamily protein |
| Phvul.007G052900.1 | AT5G54770.1 | thiazole biosynthetic enzyme, chloroplast (ARA6) (THI1) (THI4) |
| Phvul.001G101400.1 | AT5G04200.1 | metacaspase 9 |
| Phvul.005G122600.1 | AT2G27385.1 | Pollen Ole e 1 allergen and extensin family protein |
| Phvul.004G079500.1 | AT1G55370.2 | NDH-dependent cyclic electron flow 5 |
| Phvul.009G200900.1 | AT3G25690.1 | Hydroxyproline-rich glycoprotein family protein |
| Phvul.006G064100.1 | AT3G46970.1 | alpha-glucan phosphorylase 2 |
| Phvul.011G148900.1 | AT5G54190.1 | protochlorophyllide oxidoreductase A |
| Phvul.005G131000.1 | AT3G45010.1 | serine carboxypeptidase-like 48 |
| Phvul.008G257200.1 | AT2G30150.1 | UDP-Glycosyltransferase superfamily protein |
| Phvul.005G062000.1 | AT2G01590.1 | chlororespiratory reduction 3 |
| Phvul.005G108200.1 | AT4G10270.1 | Wound-responsive family protein |
| Phvul.008G100600.1 | AT2G40370.1 | laccase 5 |
| Phvul.010G073800.1 | AT2G38080.1 | Laccase/Diphenol oxidase family protein |
| Phvul.009G152200.1 | AT1G77580.2 | Plant protein of unknown function (DUF869) |
| Phvul.008G237500.1 | AT5G37600.1 | glutamine synthase clone R1 |
| Phvul.007G078600.1 | na | na |
| Phvul.011G056500.1 | AT3G45140.1 | lipoxygenase 2 |
| Phvul.001G083000.1 | AT5G13930.1 | Chalcone and stilbene synthase family protein |
| Phvul.005G084700.1 | AT1G08640.1 | Chloroplast J-like domain 1 |
| Phvul.001G174000.1 | AT5G02830.1 | Tetratricopeptide repeat (TPR)-like superfamily protein |
| Phvul.009G160600.1 | AT5G51010.1 | Rubredoxin-like superfamily protein |
| Phvul.010G049600.1 | AT3G48110.1 | glycine-tRNA ligases |
| Phvul.009G123900.1 | AT4G08290.1 | nodulin MtN21 /EamA-like transporter family protein |
| Phvul.005G108300.1 | AT4G10270.1 | Wound-responsive family protein |
| Phvul.008G290100.1 | AT3G22150.1 | Tetratricopeptide repeat (TPR)-like superfamily protein |
| Phvul.005G015900.1 | AT5G07050.1 | nodulin MtN21 /EamA-like transporter family protein |
| Phvul.007G150700.1 | AT2G37220.1 | RNA-binding (RRM/RBD/RNP motifs) family protein |
| Phvul.004G051600.1 | AT5G17920.1 | Cobalamin-independent synthase family protein |
| Phvul.003G131400.1 | AT5G20630.1 | germin 3 |
| Phvul.006G139000.1 | na | na |
| Phvul.006G178300.1 | AT1G28680.1 | HXXXD-type acyl-transferase family protein |
| Phvul.008G288600.1 | AT3G07750.1 | 3\'-5\'-exoribonuclease family protein |
| Phvul.007G021100.1 | AT2G35130.1 | Tetratricopeptide repeat (TPR)-like superfamily protein |
| Phvul.009G008100.1 | AT1G20850.1 | xylem cysteine peptidase 2 |
| Phvul.009G222700.1 | AT5G20860.1 | Plant invertase/pectin methylesterase inhibitor superfamily |
| Phvul.006G204500.1 | AT5G19473.1 | RPM1-interacting protein 4 (RIN4) family protein |
| Phvul.002G211800.1 | na | na |
| Phvul.003G154600.1 | AT5G17420.1 | Cellulose synthase family protein |
| Phvul.010G156300.1 | AT3G15840.1 | post-illumination chlorophyll fluorescence increase |
| Phvul.007G106300.1 | AT2G45550.1 | cytochrome P450, family 76, subfamily C, polypeptide 4 |
| Phvul.008G219700.1 | AT2G41430.1 | dehydration-induced protein (ERD15) |
| Phvul.008G257100.1 | na | na |
| Phvul.003G136900.1 | AT4G29720.1 | polyamine oxidase 5 |
| Phvul.003G123500.1 | AT4G05320.2 | polyubiquitin 10 |
| Phvul.008G081200.1 | AT5G01930.1 | Glycosyl hydrolase superfamily protein |
| Phvul.009G008200.1 | AT4G35350.1 | xylem cysteine peptidase 1 |
| Phvul.007G147900.1 | AT2G36885.1 | na |
| Phvul.001G242100.1 | AT2G40100.1 | light harvesting complex photosystem II |
| Phvul.004G076100.1 | AT3G14470.1 | NB-ARC domain-containing disease resistance protein |
| Phvul.003G261900.1 | AT3G51240.1 | flavanone 3-hydroxylase |
| Phvul.004G123100.1 | AT5G51890.1 | Peroxidase superfamily protein |
| Phvul.002G140400.1 | AT2G35410.1 | RNA-binding (RRM/RBD/RNP motifs) family protein |
| Phvul.008G280300.1 | AT4G37000.1 | accelerated cell death 2 (ACD2) |
| Phvul.006G140300.1 | AT4G17220.1 | microtubule-associated proteins 70-5 |
| Phvul.009G090100.1 | AT4G18780.1 | cellulose synthase family protein |
| Phvul.002G084500.1 | AT1G14220.1 | Ribonuclease T2 family protein |
| Phvul.009G256600.1 | na | na |
| Phvul.003G238700.1 | AT2G23170.1 | Auxin-responsive GH3 family protein |
| Phvul.005G002400.1 | AT5G25120.1 | ytochrome p450, family 71, subfamily B, polypeptide 11 |
| Phvul.008G100500.1 | AT5G60020.1 | laccase 17 |
| Phvul.004G171700.1 | AT2G15690.1 | Tetratricopeptide repeat (TPR)-like superfamily protein |
| Phvul.002G326700.1 | AT5G52450.1 | MATE efflux family protein |
| Phvul.009G242700.1 | AT5G44030.1 | cellulose synthase A4 |
| Phvul.010G065600.1 | AT5G01600.1 | ferretin 1 |
| Phvul.007G214200.1 | AT3G62410.1 | CP12 domain-containing protein 2 |
| Phvul.008G271700.1 | AT4G30880.1 | Bifunctional inhibitor/lipid-transfer protein/seed storage 2S albumin superfamily protein |
| Phvul.003G147700.1 | AT5G57560.1 | Xyloglucan endotransglucosylase/hydrolase family protein |
| Phvul.006G065300.1 | AT2G29630.1 | thiaminC |
| Phvul.008G239900.1 | AT1G65930.1 | cytosolic NADP+-dependent isocitrate dehydrogenase |

*na-indicates gene without annotations or without Arabidopsis homolog*
